# Cost Analysis and Supply Utilization of Laparoscopic Cholecystectomy

**DOI:** 10.1155/2018/7838103

**Published:** 2018-12-10

**Authors:** Trishul Kapoor, Sean M. Wrenn, Peter W. Callas, Wasef Abu-Jaish

**Affiliations:** ^1^Mayo Clinic, Rochester, MN, USA; ^2^University of Vermont Medical Center, Department of Surgery, Burlington, VT, USA

## Abstract

Laparoscopic cholecystectomy (LC) is one of the highest volume surgeries performed annually. We hypothesized that there is a statistically significant intradepartmental cost variance with supply utilization variability amongst surgeons of different subspecialty. This study sought to describe laparoscopic cholecystectomy cost of care among three subspecialties of surgeons. This retrospective observational cohort study captured 372 laparoscopic cholecystectomy cases performed between June 2015 and June 2016 by 12 surgeons divided into three subspecialties: 2 in bariatric surgery (BS), 5 in acute care surgery (ACS), and 5 in general surgery (GS). The study utilized a third-party software, Surgical Profitability Compass Procedure Cost Manager and Crimson System (SPCMCS) (The Advisory Board Company, Washington, DC), to stratify case volume, supply cost, case duration, case severity level, and patient length of stay intradepartmentally. Statistical methods included the Kruskal-Wallis test. Average composite supply cost per case was $569 and median supply cost per case was $554. The case volume was 133 (BS), 109 (ACS), and 130 (GS). The median intradepartmental total supply cost was $674.5 (BS), $534 (ACS), and $564 (GS) (P<0.005). ACS and GS presented with a higher standard deviation of cost, $98 (ACS) and $110 (GS) versus $26 (BS). The median case duration was 70 min (BS), 107 min (ACS), and 78 min (GS) (P<0.02). The average patient length of stay was 1.15 (BS), 3.10 (ACS), and 1.17 (GS) (P<0.005). Overall, there was a statistically significant difference in median supply cost (highest in BS; lowest in ACS and GS). However, the higher supply costs may be attenuated by decreased operative time and patient length of stay. Strategies to reduce total supply cost per case include mandating exchange of expensive items, standardization of supply sets, increased price transparency, and education to surgeons.

## 1. Introduction

With an annual incidence of over 500,000 per year, laparoscopic cholecystectomy (LC) is one of the most common abdominal surgical procedures performed in the United States [1–6]. In any surgery, a large part of total costs can be attributed to consumable supplies and it is essential for health care organizations to understand costs of high volume surgical procedures [4, 7]. This study sought to compare laparoscopic cholecystectomy cost among three subspecialties at a large academic medical center. The primary outcome was laparoscopic cholecystectomy cost. The secondary outcomes of interest included case duration and length of stay. We hypothesize that there will be a statistically significant variation in laparoscopic cholecystectomy cost amongst three different surgical subspecialties.

## 2. Materials and Methods

The University of Vermont (UVM) Institutional Review Board (IRB) exempted this study from IRB approval because of the deidentified and administrative nature of the data.

This study was a retrospective observational cohort study capturing laparoscopic cholecystectomy performed between June 2015 and June 2016 by 12 surgeons divided into three subspecialties: bariatric, acute care surgery, and general surgery. All data in this study was obtained and reviewed on the Surgical Profitability Compass Procedure Cost Manager and Crimson System (SPCMCS) (The Advisory Board Company, Washington, DC). The inclusion criteria were all elective LC cases performed from June 2015 to June 2016. Any emergent surgical procedure performed during this time period was excluded. Any surgeon performing 5 or fewer cases within the study period was excluded. All surgical cases were identified in SPCMCS using (CPT) codes. SPCMCS cost analysis included surgeon-stratified comparisons of average supply cost, average case duration, case severity (DRG groups, estimated based on ICD based coding system), patient length of stay, and supply use analysis organized by impact rating. Impact rating is a process of identifying consumable supply items that are lead drivers in cost per case variation. Supply item sorting by impact rating is completed through composite analysis of individual item cost, quantity used per case, and utilization rate [8].

All data was collected and analyzed on Microsoft Excel (Redmond, WA, USA). Data was primarily analyzed as incidence (%), median, and interquartile ranges. Statistical methods also included Kruskal-Wallis test in Statistical Package for the Social Sciences (SPSS) software. A p-value <0.05 was considered statistically significant.

## 3. Results

**Figure 1:**
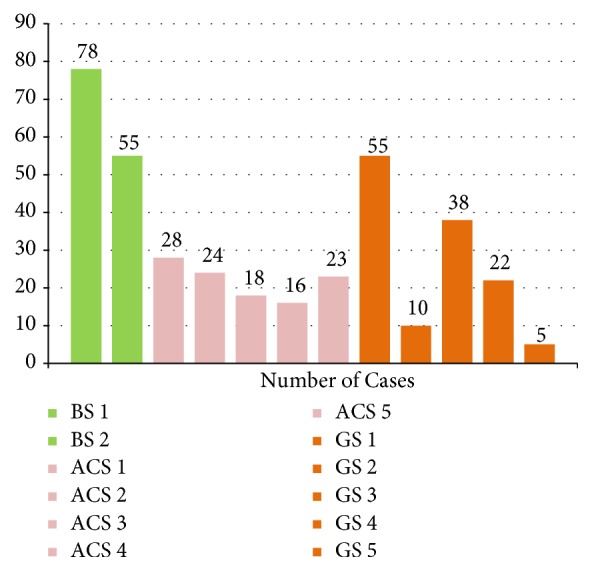
Comparison of average supply cost per case stratified by surgeon and department. The three departments analyzed include bariatric surgery (n=2), acute care surgery (n=5), and general surgery (n=5).

**Figure 2:**
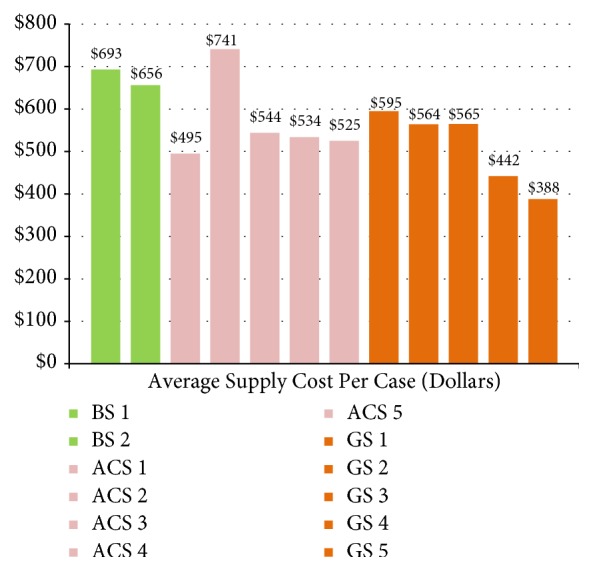
Comparison of case volume stratified by surgeon and department. The three departments analyzed include bariatric surgery (n=2), acute care surgery (n=5), and general surgery (n=5).

The case volume distribution was 133 (BS), 109 (ACS), and 130 (GS). The intradepartmental division-stratified case volume per surgeon is presented in Figure 1. While accounting for month-to-month fluctuations in mean supply cost, the average composite supply cost per case for the study period was $569 USD across all divisions within the department of surgery. The intradepartmental division-stratified average composite supply cost per surgeon is presented in Figure 2. Additional supply cost analyses are provided in Table 1. The mean supply cost in the bariatric surgery division was $674.40 with a standard deviation of $26.16 between 2 surgeons. The mean supply cost in the acute care surgery division was $567.80 with a standard deviation of $98.54 between 5 surgeons. The mean supply cost in the general surgery division was $529.00 with a standard deviation of $110.90 between 5 surgeons. These intradepartmental variations in supply cost were statistically significant (P<0.005).

The average case duration in the bariatric surgery division was 70 minutes with a standard deviation of 7.071 minutes between 2 surgeons. The average case duration in the acute care surgery division was 104.6 minutes with a standard deviation of 21.04 minutes between 5 surgeons. Lastly, the average case duration in the general surgery division was 75.8 minutes with a standard deviation of 5.07 minutes between 5 surgeons. The mean case duration distribution was statistically significant (P<0.02) and additional analyses are provided in Table 2. There was no statistically significant difference in mean case severity level (P<0.7) between bariatric surgeons (1.6), acute care surgeons (1.9), and general surgeons (1.9). However, there was a statistically significant difference (P<0.001) in average patient length of stay between the three intradepartmental divisions: 1.15 in bariatric surgery, 3.1 in acute care surgery, and 1.17 in general surgery.

Across all intradepartmental divisions, “Trocar Bladeless with Handle 11MM XCEL” by Ethicon was the surgical supply unit with the highest impact rating. The intradepartmental stratified cost analysis is presented in Table 3. The bariatric surgery division utilized this supply unit in the highest volume (105) with an overall total cost of $14,189. However, the acute care surgery division barely utilized this supply unit; only one supply unit was used during the entire study period. The second most high impact rating was the “Pack Lap Chole CDS” (Table 4), which was utilized nearly universally within the ACS and BS divisions and in half the GS cases. The total cost for this pack across all departments was $37,137 within the study period. ($121 per case).

## 4. Discussion

This study comparatively analyzed and evaluated surgeon-specific consumable supply cost and utilization for laparoscopic cholecystectomy cases performed at a large academic medical center. Amongst the three intradepartmental divisions, general surgeons had the lowest mean total supply cost and shortest average patient length of stay for laparoscopic cholecystectomies, compared to BS and ACS. What is less clear is how much these supply cost differences might affect differences in length of stay or case duration (both significant) or whether these are due to unrelated differences in case composition or clinical care.

There have been numerous studies previously reviewing strategies for maximizing the operating room profitability and efficiency. These studies have proposed a variety of methods to accomplish this goal: reducing turnover time, reorganizing patient flow, surgical team training, and standardizing surgical toolsets [9–12]. For example, Avansino et al. demonstrated a 20% average reduction in supply cost per case through standardization of operative equipment for laparoscopic appendectomies, without statistically significant changes in operative time or number of intraoperative complications. Gitelis et al. demonstrated a 10% reduction in supply costs for laparoscopic cholecystectomies through nonincentive based education of surgeons regarding cost of disposable surgical tools in a multicenter study. Similar to our study, the authors identified the most expensive consumable surgical supplies to be endomechanical staplers, clip appliers, energy devices, specimen bags, and fascial closure devices.

There are several known limitation to our study. The findings of this study may not be entirely generalizable to other healthcare institutions due the retrospective, single-institution nature of the study. In addition, there cannot be an assumption of uniformity amongst the surgeons included in this study with regard to their surgical training, technique, and capability. Although emergent cases were excluded from this dataset, the patient data considered for each of the three intradepartmental divisions (BS, ACS, and GS) was not matched or controlled. Due to the deidentified nature of the study, it would have been difficult to complete such a task.

Our study demonstrated a statistically significant intradepartmental (BS, ACS, and GS) variability in mean supply cost per case (BS>ACS>GS), patient length of stay (ACS>GS>BS), and mean case duration (ACS>GS>BS) for laparoscopic cholecystectomy. Despite the variability in supply cost, this study does not endorse the creation of uniform supply sets for LC cases. Standardization of surgical tools could potentially create opportunity for intraoperative complications or increases in case duration due to surgeon inexperience with specific supplies. Although certain studies have shown evidence of minimal overall risk with supply set standardization [12], we understand study results are largely dependent on institution-vendor pricing contracts, surgeon cohorts, and type of procedure. Instead, we propose the creation of a transparent education model for surgeons. Through such a model, surgeons will be able to engage in multidisciplinary collegial discussion and gain insight into the multivariate-dependent intradepartmental variability for supply utilization. We suggest future prospective studies should focus on the adoption of pricing educational tools to evaluate their impact.

## Figures and Tables

**Table 1:** Supply cost analysis.

	**Bariatric Surgery (n=2)**	**Acute Care Surgery (n=5)**	**General Surgery (n=5**
**Minimum**	$656	$495	$388

**25th Percentile**	$656	$510	$415

**Median**	$674	$534	$564

**75th Percentile**	$693	$642.5	$625.5

**Maximum**	$693	$741	$656

**Mean**	$674.4	$576.8	$529

**Std. Deviation**	$26.16	$98.54	$110.9

**Std. Error of Mean**	$18.50	$44.07	$49.58

**Lower 95% CI of Mean**	$439.40	$445.4	$391.3

**Upper 95% CI of Mean**	$909.6	$690.2	$666.7

**Table 2:** Case duration analysis.

	**Bariatric Surgery (n=2)**	**Acute Care Surgery (n=5)**	**General Surgery (n=5**
**Minimum**	65	83	67

**25th Percentile**	65	84	71.5

**Median**	70	107	78

**75th Percentile**	75	124	79

**Maximum**	75	133	79

**Mean**	70	104.6	75.8

**Std. Deviation**	7.071	21.04	5.07

**Std. Error of Mean**	5	9.411	2.267

**Lower 95% CI of Mean**	6.469	78.47	69.51

**Upper 95% CI of Mean**	133.5	130.7	82.09

**Table 3:** Trocar bladeless with Handle 11MM XCEL.

	**Bariatric Surgery (n=2)**	**Acute Care Surgery (n=5)**	**General Surgery (n=5**
**Total Cost**	$14,189	$107	$5,736

**Cost Per Case**	$139	$107	$144

**Total Quantity**	105	1	40

**Quantity Per Case**	1.03	1	1

**Cases**	102	1	40

**Table 4:** Pack Lap Chole CD.

	**Bariatric Surgery (n=2)**	**Acute Care Surgery (n=5)**	**General Surgery (n=5**
**Total Cost**	$15,967	$12,943	$8,225

**Cost Per Case**	$121	$121	$121

**Total Quantity**	132	107	68

**Quantity Per Case**	1	1	1

**Cases**	132	107	68
